# Tin Compensation for the SnS Based Optoelectronic Devices

**DOI:** 10.1038/srep39704

**Published:** 2017-01-03

**Authors:** S. F. Wang, W. Wang, W. K. Fong, Y. Yu, C. Surya

**Affiliations:** 1 Department of Electronic and Information Engineering the Hong Kong Polytechnic University, Hong Kong, China

## Abstract

In this paper we report the growth of high quality SnS thin films with good crystallinity deposited on two-dimensional (2D) mica substrates. It is believed that the 2D nature of SnS, with strong intra-layer covalent bonds and weak inter-layer van der Waals interactions, is responsible for its relative insensitivity to lattice mismatch. We also investigated the reduction of Sn vacancies in the material using Sn-compensation technique during the material growth process. The experimental results clearly demonstrated substantial enhancements in the electrical and structural properties for films deposited using Sn-compensation technique. A mobility of 51 cm^2^  V^−1^ s^−1^ and an XRD rocking curve full width at half maximum of 0.07° were obtained. Sn-compensated SnS/GaN:Si heterojunctions were fabricated and significant improvement in both the *I-V* characteristics and the spectral responsivities of the devices were characterized.

Tin monosulfide (SnS) is touted as a potential cost-effective optoelectronic and electronic material^1^,^2^. It has a simple molecular structure with two environmentally benign elements. The two-dimensional (2D) structure of SnS is of particular interest due to the fact that the material consists of strong fully saturated intralayer covalent bonds, but across the unit layers the interaction is dominated by weak van der Waals force^3^,^4^. Consequently, lattice match will be a less significant factor affecting the crystallinity of the SnS thin films. This property is being exploited for the growth of high-quality SnS thin films and heterojunctions for optoelectronic applications.

SnS films can be synthesized by various techniques, such as chemical vapor deposition^5^,^6^, thermal evaporation^7^,^8^, sputtering^9^, and atomic layer deposition^10^. However, the SnS films prepared by the methods above typically exhibit disordered growth orientations in addition to a polycrystalline structure, resulting in significant material defects arising from high concentration of pinholes and grain boundaries^8^,^11^. In this work, van der Waals (vdW) epitaxial growth by molecular beam epitaxy (MBE) technique was employed to produce high SnS films with high crystallinity which has strong impact on the electrical properties of the material^1^,^2^. High concentration of material defects will lead to the degradation of the electrical properties of the film and, thereby, severely affecting the application of the material in both optoelectronic and electronic devices^1^.

Successful application of SnS thin films in practical devices demands reduction of the material defect density which may serve as traps for the free carriers and have significant impact on the optoelectronic properties of the film and thereby resulting in substantial degradations in the performances of the devices. In this work, we focus on the experimental investigations of the impact of the growth parameters on the trap density of the film. In particular, we performed detailed studies of Sn-compensation on the properties of the material and the impact of the process on the performance of SnS-based optoelectronic devices. Our results demonstrate significant enhancements in the structural and electrical properties of the film and in the photocurrent output of the SnS/GaN:Si heterojunction device due to Sn compensation.

## Experiment and Results

The SnS films were prepared in an SVT 35N MBE system, which provides a highly versatile research tool that enables one to systematically vary the growth parameters with high repeatability. This facilitates systematic optimization as well as the understanding of the impact of different experimental parameters on the properties of the film. It is envisaged that once the optimal growth parameters have been determined, large-scale production of the material may be achieved by the more cost-effective thermal evaporation technique. An SnS compound source (American Elements), with 4N purity, was used for the growth of the material. Compound SnS source was chosen over elemental Sn and S sources due to the ease in controlling the sublimation rate of the compound SnS, using a conventional K-cell, compared to the elemental S source which has a high vapor pressure and may significantly affect the operation of the ultra-high vacuum chamber. A hot-lip crucible was used for the sublimation of the SnS source to avoid material condensation at the crucible opening. Mica substrate was used for the growth of the SnS films. Compared to reported results of SnS layers deposited on glass or SiO_2_^2^,^8^,^12^, thin SnS layers deposited on 2D mica demonstrate significant enhancement in the crystallinity. We exploit the fact that mica also possesses a 2D structure which ensures a weak interaction between SnS and the substrate surface and thereby significantly reduces the impact of the lattice mismatch on the crystallinity of the film. The 2D nature of mica also facilitates easy cleavage of the substrate and provides an atomically flat, electrically insulating, and optically transparent surface for the vdW epitaxial growth of SnS^13^,^14^,^15^. The temperature ramp rate of the K-cell was set at 10 °C/min with the lip and base temperatures for SnS deposition being 570 °C and 470 °C, respectively. The substrate temperature was systematically optimized and the results are summarized in Table 1S in the supplementary information. From the experimental data it is observed that the optimal substrate temperature is 270 °C, at which the narrowest rocking curve full width at half maximum (FWHM) was obtained. A strong re-evaporation occurs when the substrate temperature is higher than 300 °C, based on the film thickness measurement.

The structural property of SnS was examined by characterizing the high resolution XRD using a Rigaku Smartlab 9 kW X-ray diffractometer, equipped with a Cu-K_α1_ radiation source (λ = 1.5406 Å) and a two-crystal Ge(220) two-bounce hybrid monochromator. The XRD 2θ-ω pattern and rocking curve of SnS grown at 270 °C was shown in previous work^1^. Only the diffractions from the family of SnS {002} planes were observed, suggesting that the SnS film stacks vertically on the mica substrate with a highly c-axis oriented direction. The rocking curve measured on the SnS (004) plane showed a peak FWHM of 0.101°, implying an excellent crystallinity and demonstrating the advantage of the vdW epitaxial growth.

**Figure 1:**
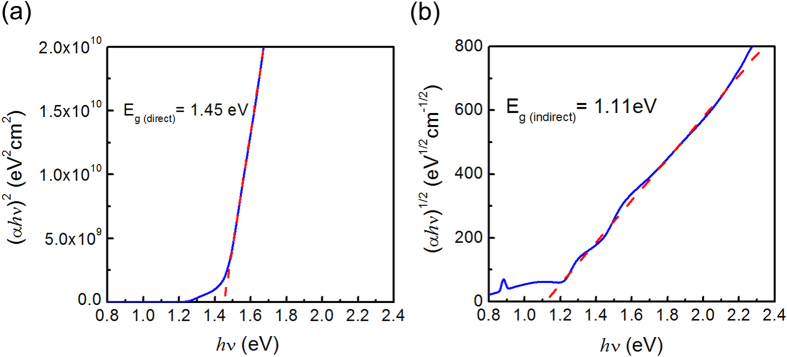
The Tauc plots for SnS film: (**a**) (*αhν*)^2^ and (**b**) (*αhν*)^1/2^ as a function of the photon energy.

The SnS optical property was studied by measuring the SnS film transmission and reflection spectra, recorded on a Hitachi U-4100 spectro-photometer. The absorption coefficient, *α*, was calculated from the optical transmittance and reflectance by using the equation^16^ below


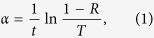


where *t* is the film thickness, *R* is the reflectance and *T* is the transmittance of the film. The optical band gap, *E*_*g*_, is estimated using the relation


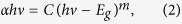


where *hv* is the photon energy, *C* is a constant, and *m* is a number related to the electron transition process. It is known that the values of *m* for allowed direct and indirect transitions are 1/2 and 2, respectively^17^,^18^. Figure 1 shows the Tauc plots for SnS films grown on the mica substrate, and the direct and indirect optical band gaps of the SnS film are found to be 1.45 eV and 1.11 eV, respectively.

**Figure 2:**
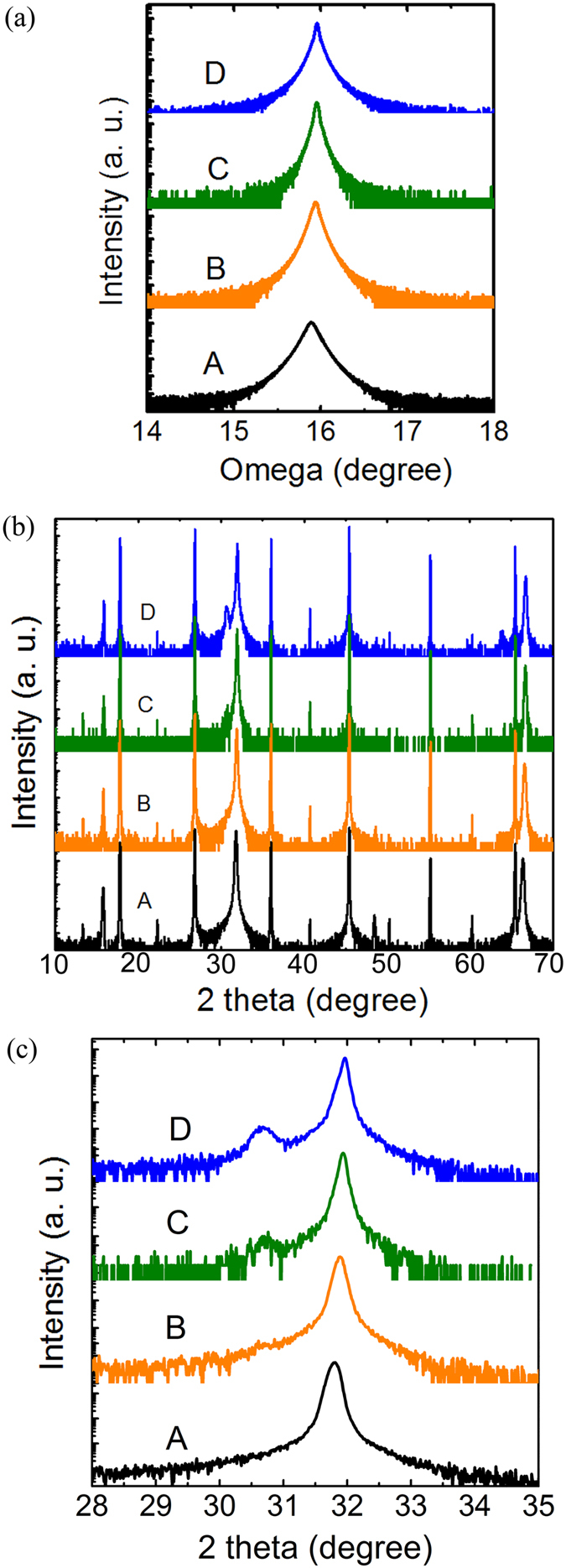
The XRD diffraction patterns of SnS film with different Sn compensation source temperature. (**a**) shows the rocking curve of SnS (004) plane; (**b**) and (**c**) are the 2θ-ω scans in the range of 10° to 70° and 28° to 35°, respectively. (A: control sample without Sn compensation; B: *T*_Sn_ = 730 °C; C: *T*_Sn_ = 750 °C; D: *T*_Sn_ = 800 °C).

The SnS electrical property was studied using a Bio-Rad 5500 four-probe Hall measurement system. The results indicate a hole concentration of 4 × 10^17^ cm^−3^ with a Hall mobility of 31 cm^2^/Vs. The p-type conductivity is attributed to the presence of Sn vacancies, which act as electron traps in the film resulting in the degradation of the structural and electrical properties of the films. To reduce the Sn vacancies in the material, a separate elemental Sn source (5N Sn pellets from American Elements) was used to suppress the formation of the Sn vacancies and to control the film electrical property. The Sn compensation source temperature was systematically varied between 700 °C and 800 °C to facilitate tuning of the SnS properties. Figure 2 shows the XRD results of the SnS films on mica at different Sn compensation source temperatures. Figure 2(a) shows the XRD rocking curves as a function of Sn compensation source temperature. The FWHMs of SnS, with Sn compensation source temperatures of 730 °C, 750 °C and 800 °C, are found to be 0.069°, 0.050° and 0.043°, respectively, whereas the FWHM of the control sample without the Sn compensation was only 0.118°. This indicates that the Sn vacancies are reduced and gives a better SnS (001) texture quality and the crystallinity. As the compensating Sn atoms occupy the Sn vacancy sites, they form chemical bonds to the neighbor sulfur atoms with dangling bonds, making the crystal more and more perfect and thus enhancing the film quality. As shown in Fig. 2(b) and (c), as the Sn compensation source temperature increased above 750 °C, an extra peak located at 2θ = 30.6° was observed and this peak intensity was enhanced when the Sn compensation source temperature was further increased. This peak is associated with the Sn (200) plane diffractions, according to the PDF database (JCPDS Card No. 04–0673). Thus, Sn segregation occurred when too high the Sn compensation flux was applied.

**Figure 3:**
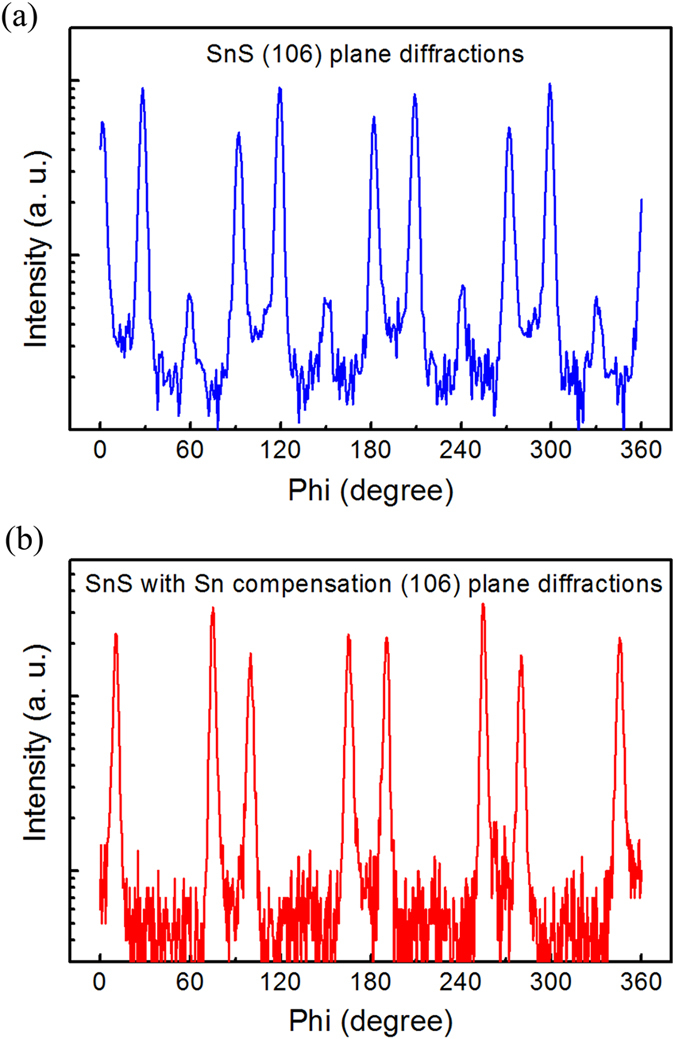
The XRD of SnS (106) in-plane phi scans of (**a**) the SnS control film without Sn compensation on mica substrate, and (**b**) the SnS film prepared with Sn compensation at *T*_*Sn*_ = 750 °C.

The XRD out-of-plane scans demonstrated an enhancement in the alignment of Sn compensated SnS crystals along the vertical stacking direction. To examine the lateral growth of SnS, the XRD in-plane phi scans were conducted on the SnS (106) planes. As shown in Fig. 3(a), eight major peaks and 4 minor peaks, separated by about 30°, are observed in the SnS control sample, implying that SnS crystals grow laterally on the mica substrate along six directions with 30° intervals. The crystal coalescence of this multi-orientation growth results in high concentration of grain boundaries and material defects. Using Sn compensation technique, the minor lateral orientations are substantially suppressed as indicated by the extinction of the minor peaks in Fig. 3(b). This suppression of the minor lateral orientations suggests enhancement in the lateral growth alignment of SnS crystals. The Sn compensation has almost no effect on the optical property of SnS (see Figure 1S in the supplementary information).

**Figure 4:**
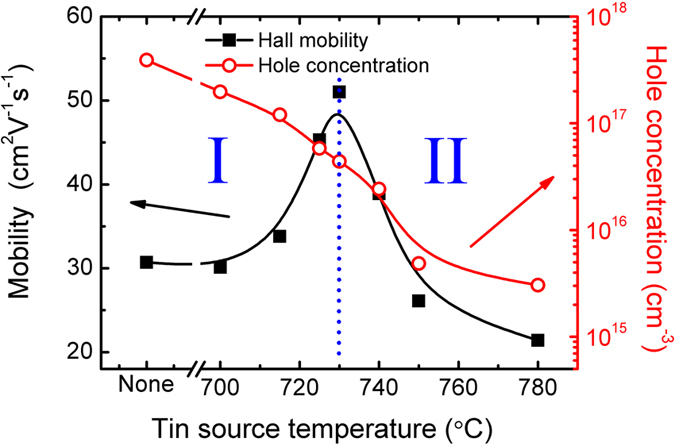
The variations of the Hall mobility and the hole concentration at different Sn compensation source temperatures.

The compensating Sn atoms incorporated into the film not only enhance the film quality, but also passivate the sulfur dangling bonds in the film. Figure 4 illustrates the variation of the hole concentration and the Hall mobility as a function of *T*_*Sn*_. It is observed that the SnS hole concentration decreased by over two orders of magnitude from 4 × 10^17^ cm^−3^ until beyond the Bio-Rad 5500 Hall system measurement limit. The increase in *T*_*Sn*_ stipulates a corresponding increase in the amount of Sn atoms incorporated into the film, suggesting that the intrinsic Sn vacancies in the film are effectively eliminated by the extrinsic Sn atoms. A peak mobility of 51 cm^2^ V^−1^ s^−1^ is obtained at the hole concentration of 4.4 × 10^16^ cm^−3^. Thus it is clear that the compensating Sn atoms enable the tuning of the film electrical property as well as the improvement in the crystal alignment, both vertically and laterally. However, the drop in the mobility at higher Sn compensation source temperature is attributed to the Sn metal segregation in the film as shown in Fig. 2(b). The Hall measurement data in Fig. 4 can be divided into two regions: Region I for *T*_*Sn*_ ≤ 730 °C; and Region II for *T*_*Sn*_ ≥ 730 °C. In Region I, by introducing the compensating Sn atoms into the film, a systematic lowering in the hole concentration was observed, indicative of the fact that the Sn atoms from the compensating source mostly occupied the vacancy states. The lowering in the number of Sn vacancies will benefit the carrier mobility, thus leading to an increase in the mobility in Region I in the Hall measurement. As more and more Sn atoms from the compensation source are incorporated into the material, excessive Sn atoms will form Sn clusters in the film, as observed in Fig. 2(c) for the Sn compensated samples at *T*_*Sn*_ ≥ 750 °C. This is accompanied by the reduction in the Hall mobility, indicating the increase in scattering by the Sn clusters. Continuous decrease in the hole concentration was observed in Region II, as the Sn vacancy filling process continued. Thus, Sn compensation has to be optimized carefully in order to enhance the feasibility of optoelectronic applications of the SnS thin films.

**Figure 5:**
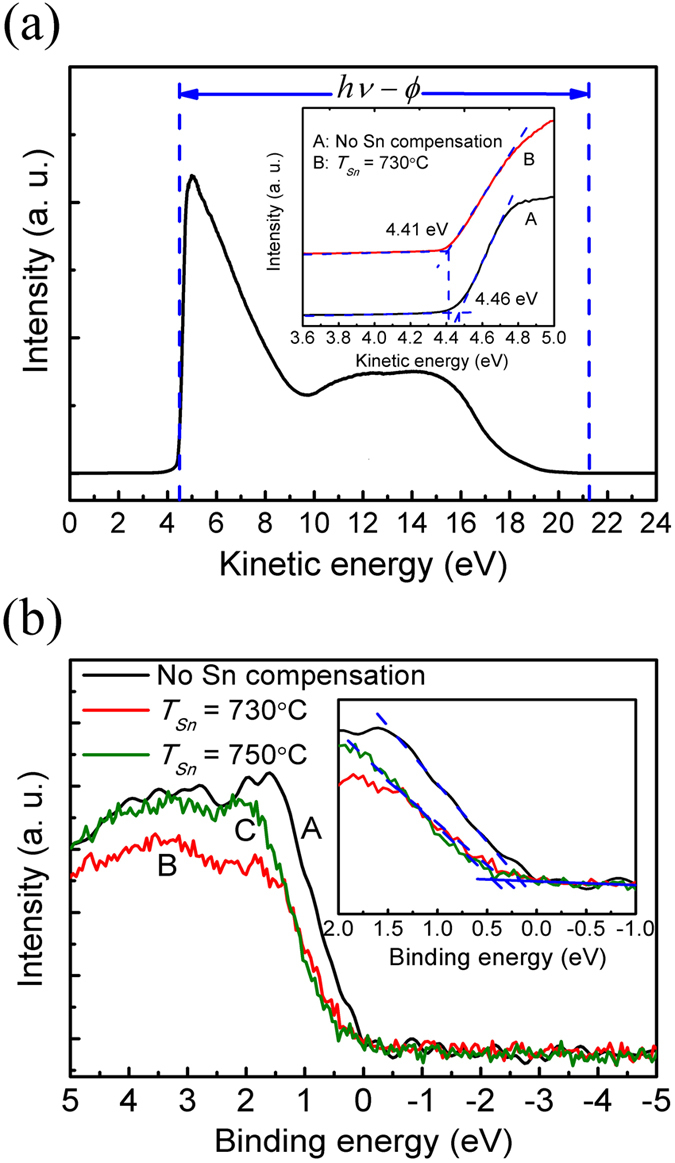
The photoemission spectroscopy measurements of the SnS films. (**a**) UPS for SnS samples without Sn compensation (denoted as A) and with Sn compensation at *T*_*Sn*_ = 730 °C (denoted as B); (**b**) XPS for SnS samples without Sn compensation (denoted as A), and with Sn compensation at *T*_*Sn*_ = 730 °C (denoted as B) and *T*_*Sn*_ = 750 °C (denoted as C).

To investigate the effects of Sn compensation on the optoelectronic properties of SnS-based device, an SnS based *p-n* heterojunction was fabricated using MBE technique. The final device demands proper band alignment between the *n*- and *p*-type materials. The electron affinity and work function of as-grown SnS were examined by ultraviolet photoelectron spectroscopy (UPS) and X-ray photoelectron spectroscopy (XPS). The UPS was conducted in an ESCALAB 250 spectrometer using He I radiation (*hν* = 21.22 eV) as the UV source, with energy step size of 20 meV. The XPS was recorded by a Shenyang SKL-12 electron spectrometer using a non-monochromatic Al Kα radiation (*hν* = 1486.6 eV) as the excitation source. It was equipped with a VG CLAM 4 MCD electron energy analyzer. The base pressure in the analysis chamber was about 2 × 10^−9^ mbar. All the XPS spectra were calibrated by adjusting the internal standard C 1 s core level position to 284.8 eV^19^,^20^. As shown in Fig. 5(a), the work functions of SnS film without Sn compensation and with Sn compensation at *T*_*Sn*_ = 730 °C are found to be 4.46 eV and 4.41 eV, respectively, by linearly extrapolating the low kinetic energy leading edge of the UPS spectrum to the background base line. The SnS valence band maximum (VBM) position relative to the Fermi edge is determined from the intersection of the linear fitting to the leading edge of the XPS spectrum and the background, as shown in Fig. 5(b). The difference between the VBM and Fermi edge for SnS samples without Sn compensation, and with Sn compensation at *T*_*Sn*_ = 730 °C and *T*_*Sn*_ = 750 °C are extracted to be 0.17 eV, 0.28 eV and 0.34 eV, respectively, which are consistent with the observed *p*-type conductivity of the film. Moreover, it is deduced from the XPS spectra that the Fermi level of the Sn-compensated samples shifts upwards, which is consistent with the slight drop in work function extracted from the UPS spectra. Combining the energy band gap, work function, and the VBM position, the SnS electron affinity is determined to be 3.52 eV, which is smaller than that of most the commonly used *n*-type layers, such as AZO, FTO, ITO (~4.35 eV)^21^.

**Figure 6:**
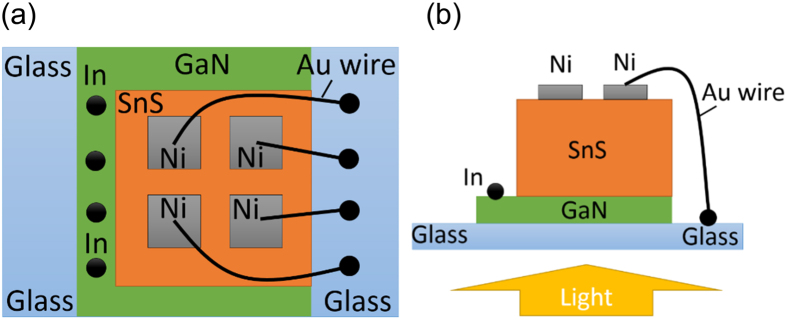
The SnS/GaN:Si heterojunction device structure. (**a**) Top-view; (**b**) Side view.

The relatively low electron affinity of SnS film limits the choice of n-type layer, to form a good *p-n* heterojunction optoelectronic device. To match the low electron affinity of SnS, tunable window layers such as Zn(O, S)^10^, Zn_1−x_Mg_x_O^9^, were applied by other research groups. In this work, a silicon-doped gallium nitride (GaN:Si) layer was used as the *n*-type window electrode, because of the low electron affinity value reported between 2.9 and 3.2 eV^22^. A 2 μm thick GaN:Si window layer was grown on sapphire substrate. The GaN:Si was dipped into diluted hydrochloric acid (HCl:H_2_O = 1:1) for 1 minute to remove the native oxide layer formed on the surface, followed by rinsing with deionized water prior to the transfer into the MBE chamber. The SnS film was then deposited onto the GaN:Si layer. Only the diffractions from the SnS {002} family of planes are observed in the XRD 2θ-ω scan (see Figure 2S in the supplementary information), suggesting good crystallinity for the SnS layer. A shadow mask with square openings (2 × 2 mm^2^) was used to deposit 120 nm thick Ni electrodes by E-beam evaporation technique. Indium was used to form the ohmic contact on the *n*-type GaN:Si window layer. The device was then attached to a glass substrate which enabled light to incident on the device from the glass side. The structure of the heterojunction device is shown in Fig. 6.

The valence band offset between the SnS and GaN:Si heterojunction (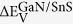) was examined by XPS measurement and is calculated by


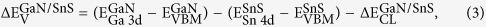


where 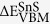 and 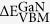 are the respective VBM positions of SnS and GaN, and 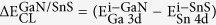 is the difference between Ga 3d (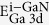) and Sn 4d (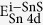) core levels at the SnS/GaN heterojunction interface. Thus, Equation (3) can be re-written as


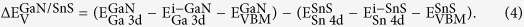


The conduction band offset of GaN:Si/SnS heterojunction (*ΔE*_*C*_) is determined as


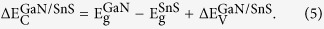


**Figure 7:**
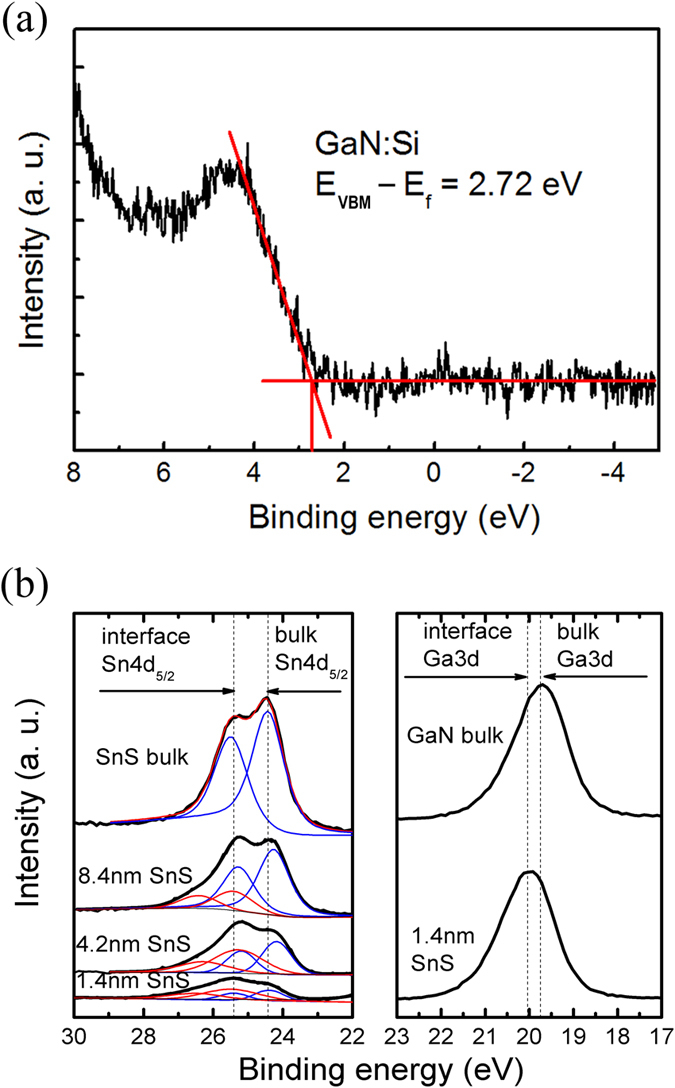
XPS spectrum of (**a**) GaN:Si substrate VBM; (**b**) peak shift between the SnS/GaN:Si interface.

**Figure 8:**
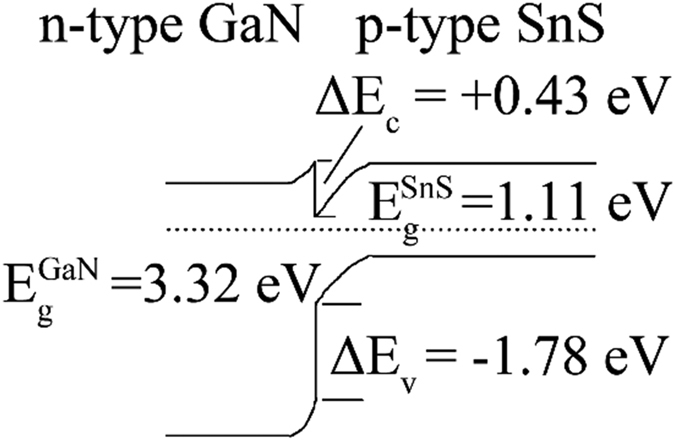
The energy band diagram of SnS/GaN:Si heterojunction.

The XPS spectra of the GaN:Si substrate and the peak shift between the SnS/GaN:Si in the interface are shown in Fig. 7. The GaN:Si VBM was determined as 2.72 eV. The shift of Ga 3d between bulk and interface of GaN is deduced to be about −0.22 eV; while a similar shift of Sn 4d core level between bulk and the interface of SnS was observed to be about −1.00 eV. By substituting these values to Equation (4), 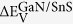 of GaN/SnS heterojunction was determined to be −1.78 eV. The GaN:Si energy band gap, 3.32 eV, used in this work is evaluated from the plot of the transmittance as a function of the incident photon energy, as shown in Figure 3S in the supplementary information. Thus 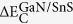 is determined to be +0.43 eV. The energy band diagram of the SnS/GaN:Si heterojunction device is illustrated as Fig. 8 based on the above findings. The work function difference between the SnS and GaN:Si is deduced to be 0.77 eV, in excellent agreement with the built-in voltage of 0.79 eV obtained from *C-V* measurement of the heterojunction device (see Figure 4S in the supplementary information).

For a SnS based heterojunction device, a “spike” in the conduction band offset (CBO) can minimize the interface recombination at the *p-n* junction as long as the spike height is kept below 0.4 eV^23^,^24^. As shown in Fig. 8, a “spike” in the CBO is formed at the SnS/GaN:Si heterostructure interface with a height of +0.43 eV, which is slightly higher than the optimal value for the Type I heterostructures^23^,^24^. In addition, the electron affinity of the GaN:Si layer is deduced as 3.09 eV, which is in good agreement with the reported values^22^.

**Figure 9:**
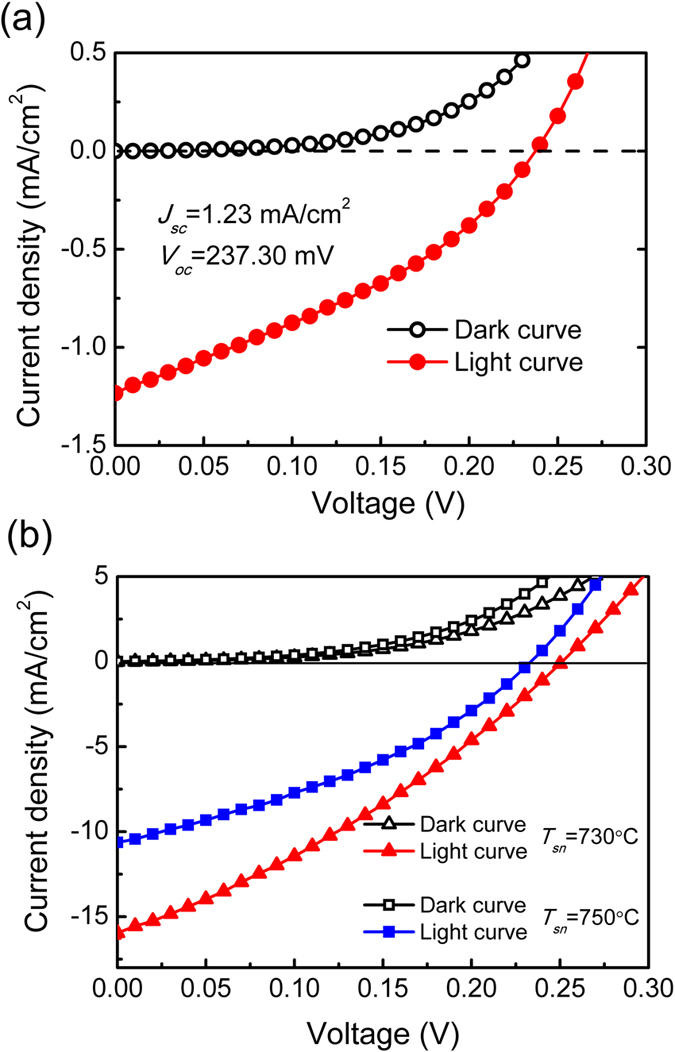
The I-V characteristics of SnS/GaN:Si heterojunction solar cells with different Sn compensation: (**a**) non-Sn compensation; (**b**) Sn compensation at *T*_Sn_ = 730 °C and *T*_Sn_ = 750 °C.

The *I-V* characteristics of SnS/GaN:Si heterojunction devices were recorded by an Agilent B1500A semiconductor device parameter analyzer. The photocurrent was measured under a simulated Air Mass 1.5 Global (AM 1.5 G) illumination, which was provided by a Newport Oriel Sol3A Solar Simulator with a 100 mW/cm^2^ radiance intensity. Figure 9 shows the *I-V* characteristics of a SnS/GaN:Si heterojunction device as a function of *T*_*Sn*_. For the devices with Sn compensation, the photo-generated current increases substantially by more than 10 times to the device without Sn compensation from 1.23 mA/cm^2^ to 15.96 mA/cm^2^. With excessive amount of Sn in the SnS film, the photocurrent density drops to 10.63 mA/cm^2^ at zero bias and the open circuit voltage also decreases slightly. From the point of view of energy band theory, when the Sn vacancies in the film are filled and the carrier concentration decreases, the Fermi level of SnS thin film will shift upwards, reflected by the slight decrease in work function deduced from the UPS spectra and the increase in (*E*_*VBM*_ − *E*_*f*_) extracted from the XPS spectra. As a result, the work function difference between the SnS and GaN will be narrowed, leading to the slight decrease in *V*_*oc*_. The observed enhancement in the photocurrent is attributed to the reduction in the trap density due to Sn vacancies by the extrinsic Sn atoms and, thereby, the enhancement in the minority carrier diffusion length in the absorbing layer. As a result, the photocurrent is greatly increased. However, with excessive compensating Sn atoms, metallic Sn crystallites can be observed in the film, as shown in Fig. 2(b). This leads to a significant degradation in the photocurrent of the device as shown in Fig. 9(b).

**Figure 10:**
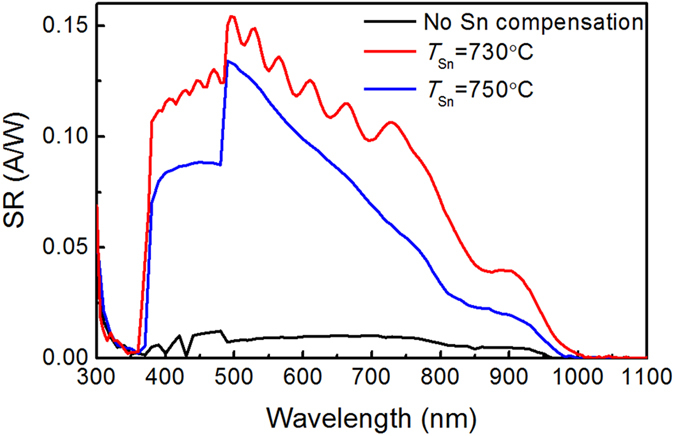
The SR spectra of SnS/GaN:Si heterojunction devices.

Figure 10 illustrates the spectrum responsivity (SR) of the devices. It is observed that without Sn compensation, the optoelectronic device SR (black curve) is very small over the entire range, suggesting that most of the photo-generated carriers cannot be extracted and collected to the external electrodes, and are probably recombined in the absorber layer due to the Sn vacancies. In contrast, by applying Sn-compensated SnS absorbers the data are indicated by the red and blue lines in Fig. 10, which demonstrate a high SR in the region between 370 and 1000 nm, originating from the SnS absorption. The device SR reaches a maximum value of 0.15 A/W at wavelength of ~500 nm when Sn compensation source temperature at 730 °C was applied. Thus the data clearly show that Sn vacancies in the SnS film act as the recombination centers and photo-generated electrons fail to be extracted from the absorber layer. This has led to significant degradation in the optoelectronic properties of the device. When the compensating Sn atoms occupy the native vacancies, the concentration of the recombination centers is significantly reduced, resulting in the enhancement in the SR in the range between 370 and 1000 nm for the heterojunctions. Excessive incorporation of Sn atoms in the SnS layer results in the segregation of Sn atoms, which leads to a degradation in the device performance.

## Conclusion

Highly textured SnS was grown on mica substrate by van der Waals epitaxy using a MBE system. It is found that applying Sn compensation during the growth can significantly improve the crystallinity. The XRD rocking curve FWHM as narrow as 0.07° was achieved by optimizing the amount of supply of compensated Sn atoms. These extrinsic Sn atoms serve to fill up the Sn vacancies, which are the origin of the recombination centers in the SnS absorbing layer.

SnS/GaN:Si heterojunction devices were fabricated to study the positive effects of the Sn compensaten SnS on the optoelectronic device photo response. Without the Sn compensation, the device spectrum response to the sunlight originates mainly from the GaN:Si side, yielding a very small photocurrent and an extremely low SR value. In contrast, when Sn compensation is applied, the device spectrum response predominantly originates from the SnS layer, reflected by the high SR in the range between 370 and 1000 nm wavelength. As a result, the photocurrent is increased by over 10 folds, from 1.23 mA/cm^2^ to 15.96 mA/cm^2^. An excessive Sn compensation, however, results in Sn segregation, degrading the film quality and thus the device performances. Detailed energy band alignment studies were also performed to elaborate the benefits of Sn compensation on the device optoelectronic performance.

## Additional Information

**How to cite this article**: Wang, S. F. *et al*. Tin Compensation for the SnS Based Optoelectronic Devices. *Sci. Rep.* **7**, 39704; doi: 10.1038/srep39704 (2017).

**Publisher's note:** Springer Nature remains neutral with regard to jurisdictional claims in published maps and institutional affiliations.

## Supplementary Material

Supplementary Information
