# Supplementary material for: Tin Compensation for the SnS Based Optoelectronic Devices
Source: Sci Rep. 2017 Jan 3;7:39704. doi: 10.1038/srep39704 (PMC5206617; doi:10.1038/srep39704)
Supplement: Supplementary Information [file srep39704-s1.doc]

**Tin Compensation for the SnS Based Optoelectronic Devices**

*S. F. Wang, W. Wang, W. K. Fong, Y. Yu, and C. Surya*^*^

Department of Electronic and Information Engineering

The Hong Kong Polytechnic University, Hong Kong, China

Email: charles.surya@polyu.edu.hk

**Supplementary Information**

S1. The XRD rocking curve FWHM of SnS thin films on the mica substrate as a function of the substrate temperature.

Table 1S. Rocking curve FWHM for SnS as a function of the growth temperature.

| **Substrate temperature (°C)** | **220** | **250** | **270** | **300** |
| --- | --- | --- | --- | --- |
| **Rocking curve FWHM (°)** | 0.236 | 0.126 | 0.101 | 0.120 |

S2. The optical properties SnS with the varying Sn compensation source temperature.


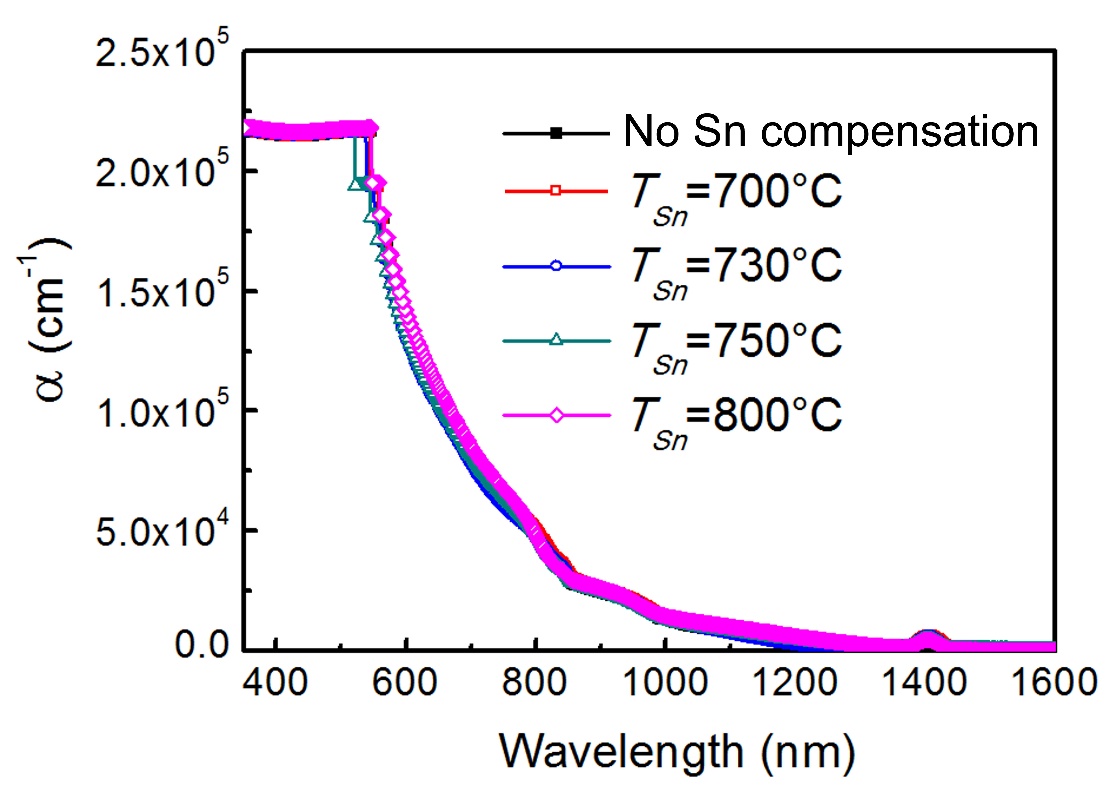


Figure 1S. The absorption coefficient, *α*, of SnS at different *T_Sn_* as a function of the incident photon wavelength.

S3. The XRD 2θ-ω scan of SnS on GaN:Si/sapphire substrate.


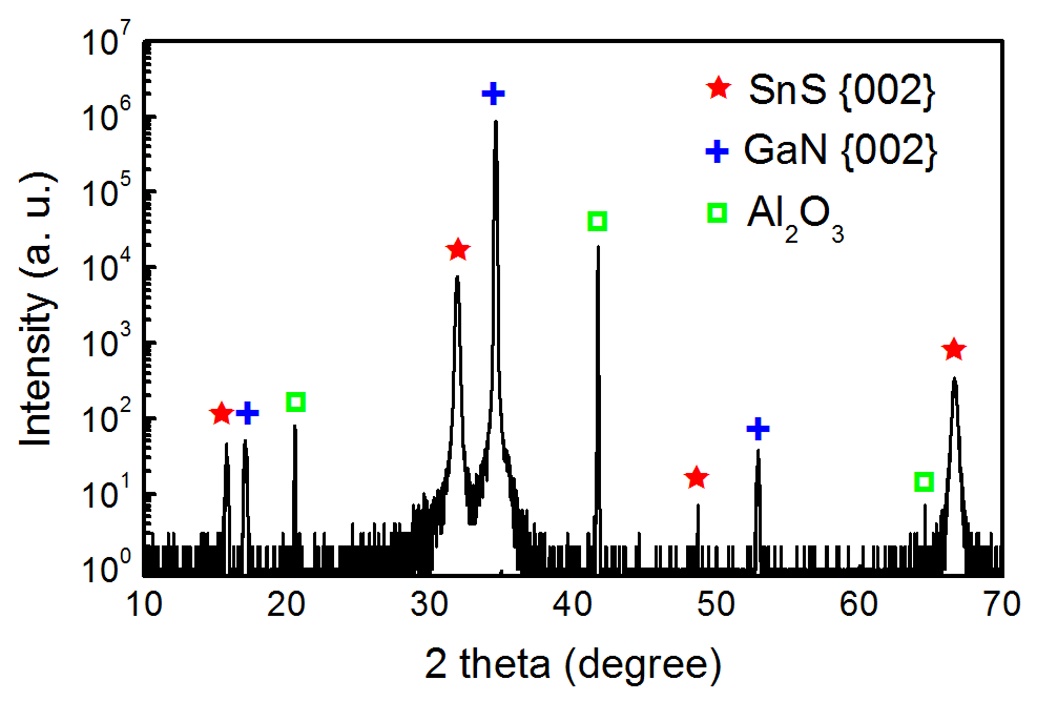


Figure 2S. The XRD 2θ-ω scan of SnS on GaN:Si/sapphire substrate. The peaks originate from the SnS {002}, GaN {002} and sapphire diffractions are labeled by the red crosses, blue diamonds and green squares, respectively.

S4. The transmittance of GaN:Si as a function of the incident photon wavelength.


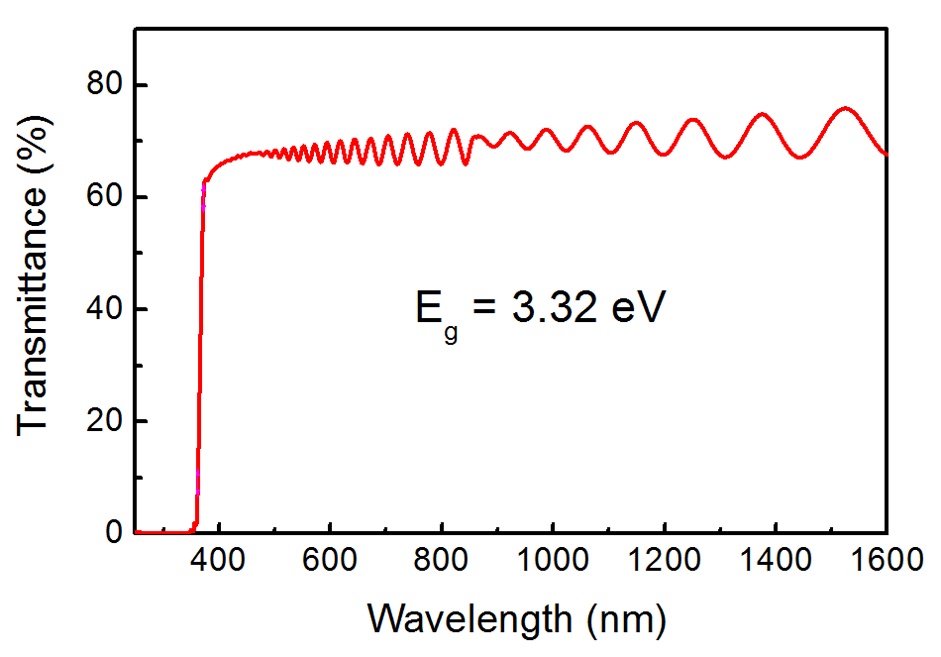


Figure 3S. The variation of the transmittance of GaN:Si on sapphire substrate as a function of the incident photon wavelength.

S5. The C-V measurement of SnS/GaN:Si heterojunction device.


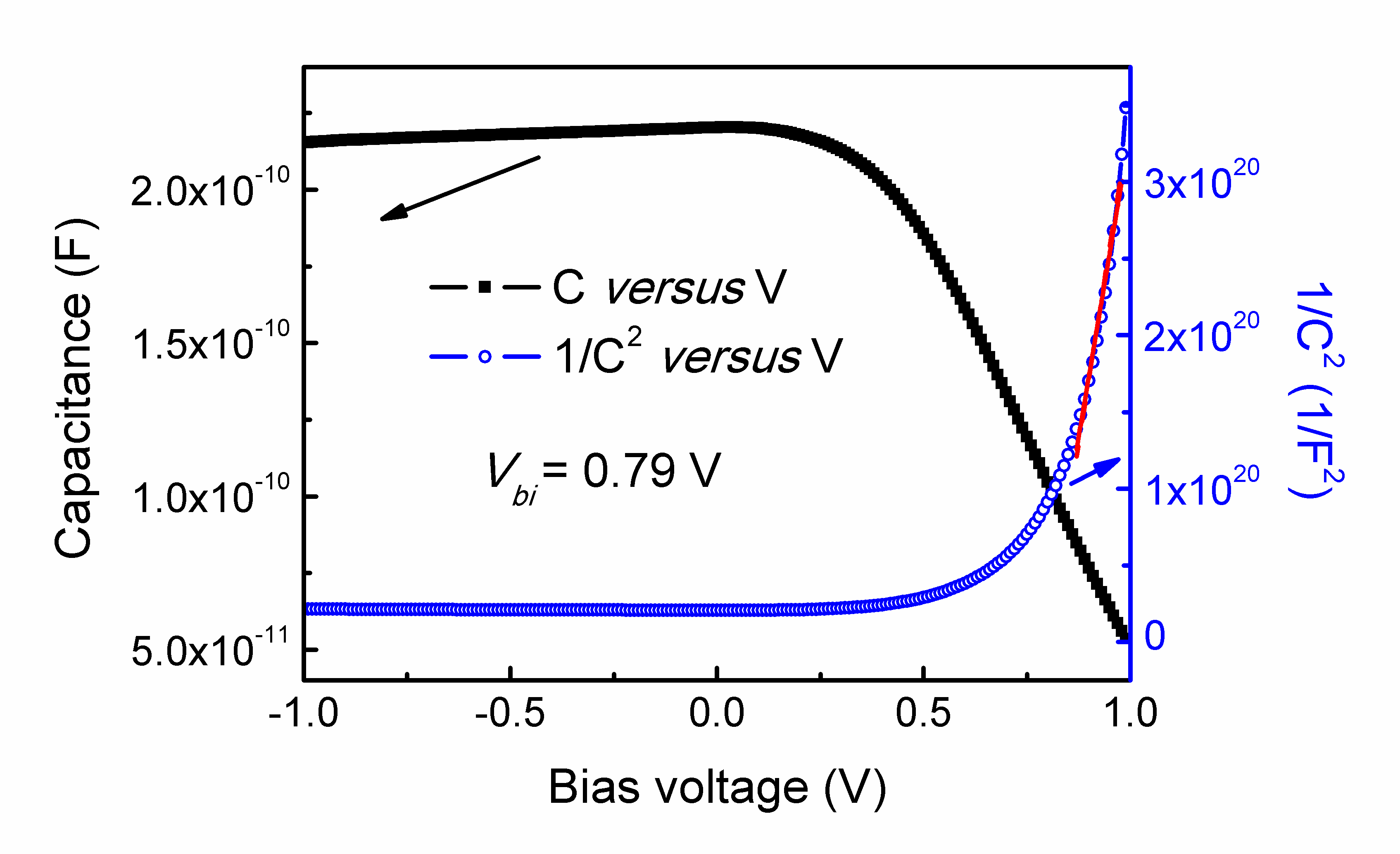


Figure 4S. The *C-V* measurement of SnS/GaN:Si heterojunction device. The built-in voltage at the heterojunction device is deduced to be 0.79 eV from the *1/C^2^* *versus* *V* curve.
